# Rapidly evolving purpuric lesions to massive hemorrhagic bullae, with rapid improvement by Prednisolone: as a coetaneous manifestation of Systemic Lupus Erythematosus: a case report

**DOI:** 10.1186/1757-1626-1-79

**Published:** 2008-08-08

**Authors:** Farzin Khorvash, Alireza Emami Naeini, Mohaddeseh Behjati, Mansoor Karimifar, Fariborz Khorvash, Koorosh Dialami

**Affiliations:** 1 Assistant Professor, Department of Infectious and Tropical Research Center, Isfahan University of Medical Sciences, Isfahan, Iran; 2 Associate Professor, Department of Infectious and Tropical Research Center, Isfahan University of Medical Sciences, Isfahan, Iran; 3 Resident, Department of Cardiology, Isfahan University of Medical Sciences, Isfahan, Iran; 4 Assistant Professor, Department of Rheumatology, Isfahan University of Medical Sciences, Isfahan, Iran; 5 Neurologist, Isfahan University of Medical Sciences, Isfahan, Iran; 6 Resident, Department of Infectious and Tropical Diseases, Isfahan University of Medical Sciences, Isfahan, Iran

## Abstract

**Background:**

Systemic lupus Erythematosus is a chronic autodestructive disease, with loss of immune tolerance to nucleic acid and other cross reactive antigens. Despite of the numerous studies, the presence of some new manifestations indicates the greater proportion of unknown data.

**Case presentation:**

Our case, is a 26-year-old female, by the chief complaint of headache, vomiting, fever and arthralgia. Some hemorrhagic ulcers in her mouth with fulminant pethechia/purpura on her limbs and buttocks were prominent. On admission, she was in hypotensive state. By the clinical suspicion to meningococcal septicemia, lumbar puncture was performed, and antibiotic therapy was started. Cerebrospinal fluid was normal. Suddenly, on the 3^rd ^day of admission, hemorrhagic bullae were evolved from those purpuric lesions. Leukocytosis, immune hemolytic anemia, thrombocytopenia and high antinuclear antibody/double stranded DNA level and hypocomplemania were present simultaneously. In skin biopsy, immune complex deposition in dermoepidermal junction was seen. The diagnosis of Systemic lupus Erythematosus was made. The patient responded well to corticosteroid therapy.

**Conclusion:**

Coetaneous manifestations are very common in Systemic lupus Erythematosus, and help the physician making differential diagnoses and proper diagnosis. The rapidly evolving hemorrhagic bulla from primary purpuric lesions, with rapid response to Prednisolone, is a rare manifestation of Systemic lupus Erythematosus, which should be considered in such a disease setting.

## Background

Systemic lupus Erythematosus (SLE) is an autodestructive disease [1], in which loss of immune tolerance to nucleic acid antigens and other cross reactive antigens has a fundamental role [2]. Despite of the numerous studies, the presence of some new manifestations indicates the greater proportion of unknown data.

## Case presentation

**Figure 1:**
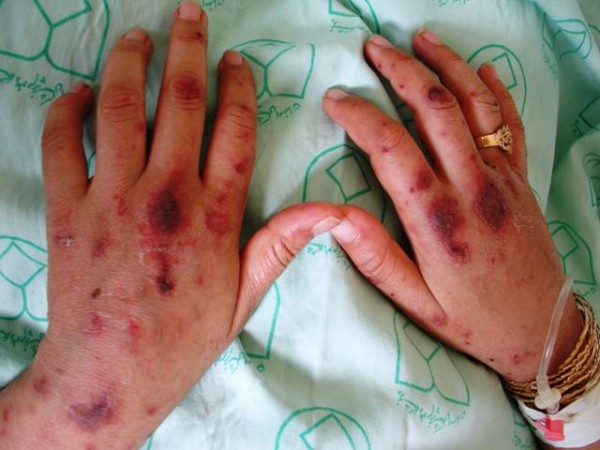
Fulminant pethechia/purpura on the limbs in the first day of admission.

**Figure 2:**
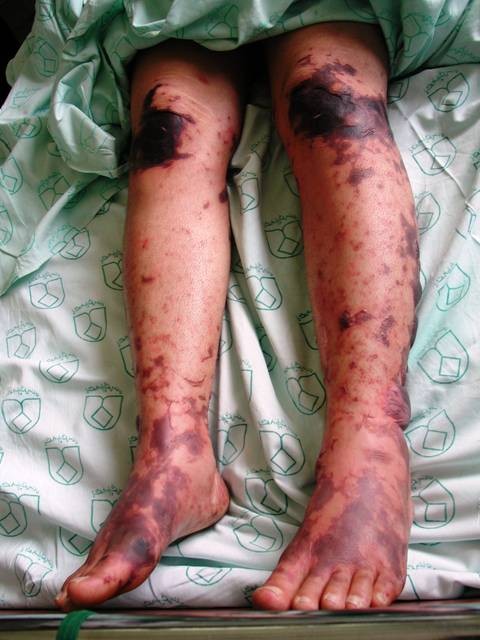
Hemorrhagic bullae on the 3^rd ^day of antibiotic therapy.

The patient was a 26-year-old female, who referred to our center, Infectious disease, Alzahra Hospital, Isfahan, Iran, by the chief complaint of headache, vomiting and fever from the day before admission. She also was complaining from arthralgia in wrists and knees, accompanied with generalized pain. Also, some hemorrhagic ulcers in her mouth and fulminant pethechia/purpura on her limbs and buttocks were prominent (Figure 1). On admission, she was in hypotensive state with some degree of agitation. A mild periorbital edema was also present. So, she was hospitalized by the clinical suspicion to meningococcal septicemia. Injection of Ceftriaxon plus Vancomycin was started empirically, and lumbar puncture (LP) performed. Despite of the normal CSF, antibiotics therapy was continued, up to getting the results of blood culture, ready. Suddenly, on the 3^rd ^day of antibiotic therapy, hemorrhagic bullae were evolved from those purpuric lesions (Figure 2). These bullae were extending beyond the previous margins. Due to the negative cultures from blood and bulla contents, antibiotics were discontinued.

The positive findings in lab data and paraclinical examination were as follow: Leukocytosis (WBC: 20100/μl), immune hemolytic anemia (Hb: 7 g/dl) plus positive direct Coobms test, thrombocytopenia (platelet: 53000/μl) and high ANA/dSDNA level. C3 and C4 level were 42 (normal range: 55–170) and 12 (normal range: 10–55), both in low levels. 3 omg/dl protein was present in the derived urine sample. Skin biopsy demonstrated some epidermal necrosis, basal layer degeneration, leukocytoclasis, dermal neutrophil infiltration, RBC extravasation plus immune complex deposition in dermoepidermal junction. By rheumatologic consultation, the diagnosis of SLE was made (fulfilling 4 criteria of 11). Cellcept was prescribed for the patient; in combination with 60 mg of daily Prednisolone. After beginning of the treatment the skin lesions was controlled but convert to disseminated necrosis. By gradual disappearance of acute phase of the disease, Anti-dSDNA and ANA were tested again, which was as high as the first checking.

## Discussion

SLE, inflammatory chronic diseases [3], with unknown etiology, is a prototype of auto immune diseases [4]. It may affect a number of organ systems in the body, in which joints, skin, kidneys and lungs are the most common involved sites [5]. Among these manifestations, skin lesions; poses a broad spectrum, usually at the active phase of disease [6] and a great interest in understanding the probable role of DNA participation, is evolved todays [7]. These skin manifestations have great diagnostic role in the diagnosis of SLE. Some of these reported manifestations are malar rashes [8], erythematous lesions [9], discoid lesions [9], non-scarring alopecia [10] and so on. But what's the importance of paying attention to these coetaneous manifestations? It's well known that improved diagnosis and treatment of SLE resulted in significant decrement of morbidity from SLE. To widen the demarcated line between alive or dead cases, a great clinical suspicion, is needed, especially on coetaneous manifestations, because of their broad spectrum and being early detectable by vision. There are also some rare coetaneous manifestations beyond the domains of physician's experience and reports. So, attention to the rare manifestations aside with the clinical and paraclinical data, afford us the increased sensitivity in the diagnosis of complicated cases with difficult diagnosis and resistant to treatment due to the wrong diagnosis.

Our case is also an interesting one with rapidly evolving skin lesions, from diffused purpuric lesions to hemorrhagic bullae. It's clear that this kind of rapidly evolving purpuric lesions to massive hemorrhagic bullae can only be seen in purpura fulminance and DIC [11], but our case was not a case of DIC. These evolving lesions had a good response to oral Prednisolone, besides of the normal PT/PTT/INR values accompanied with immune hemolytic anemia and thrombocytopenia. So, it seems that purpuric lesions with progression toward hemorrhagic bullae can be seen within the natural course of SLE. In the setting of mentioned skin lesions, SLE should be considered as a differential diagnosis, it may help the physician requesting proper paraclinical tests for proper diagnosis and treatment.

## Conclusion

Coetaneous manifestations are very common in SLE, and help the physician making differential diagnoses and proper diagnosis. The rapidly evolving hemorrhagic bulla from primary purpuric lesions, with rapid response to Prednisolone, is a rare manifestation of SLE, which should be considered in such a disease setting.

## Abbreviations

SLE: Systemic lupus Erythematosus; ANA: Antinuclear antibody; dsDNA: double stranded DNA; DIC: Disseminated Intravascular Coagulation; CBC: Cell Blood Count; CSF: Cerebro Spinal Fluid; LP: Lumbar Puncture; Hb: Hemoglobin.

## Authors' contributions

FK is corresponding author of the manuscript and infectious disease manager of the case, AEN and KD helped in infectious disease management, MB helped in writing of article, FK helped in neurologic management and MK helped in Rheumatologic management of the case. All authors read and approved the final manuscript.

## Consent

Written informed consent was obtained from the patient for publication of this case report and accompanying images. A copy of the written consent is available for review by the Editor-in-Chief of this journal.

## References

[B1] Adelman MK, Schluter SF, Robey IF, Marchalonis JJ (2007). Natural and autoantibodies to human T-cell receptor Vbeta segments: potential roles in immunomodulation. Crit Rev Immunol.

[B2] Ramanujam M, Davidson A Targeting of the immune system in systemic lupus erythematosus. Expert Rev Mol Med.

[B3] Carreño L, López-Longo FJ, González CM, Monteagudo I (2002). Treatment options for juvenile-onset systemic lupus erythematosus. Paediatr Drugs.

[B4] Casciola-Rosen L, Rosen A (1997). Ultraviolet light-induced keratinocyte apoptosis: a potential mechanism for the induction of skin lesions and autoantibody production in LE. Lupus.

[B5] Carron JD, Karakla DW, Watkins DV (1999). Focal parotid necrosis in systemic lupus erythematosus: case report and review of the literature. Oral Surg Oral Med Oral Pathol Oral Radiol Endod.

[B6] Werth VP (2005). Clinical manifestations of cutaneous lupus erythematosus. Autoimmun Rev.

[B7] Ebling FM, Hahn BH (1989). Pathogenic subsets of antibodies to DNA. Int Rev Immunol.

[B8] Balkaran BN, Roberts LA, Ramcharan J (2004). Systemic lupus erythematosus in Trinidadian children. Ann Trop Paediatr.

[B9] Shidara K, Soejima M, Shiseki M, Ohta S, Nishinarita M (2003). A case of systemic lupus erythematosus complicated with psoriasis vulgaris. Nihon Rinsho Meneki Gakkai Kaishi.

[B10] Tebbe B (2004). Clinical course and prognosis of cutaneous lupus erythematosus. Clin Dermatol.

[B11] Gamba G, Montani N, Montecucco CM, Caporali R, Ascari E (1991). Purpura fulminans as clinical manifestation of atypical SLE with antiphospholipid antibodies: a case report. Haematologica.

